# Clinical characteristics of COVID-19 associated vasculopathic diseases

**DOI:** 10.1186/s12959-023-00504-4

**Published:** 2023-05-25

**Authors:** Thiemo Greistorfer, Philipp Jud

**Affiliations:** Division of Angiology, Department of Internal Medicine, Medical University of Graz, Auenbruggerplatz 15, Graz, 8036 Austria

**Keywords:** COVID-19, Venous thromboembolism, Arterial thromboembolism, Vasculitis

## Abstract

Coronavirus disease 19 (COVID-19) has shown to be an infectious disease affecting not only of the respiratory system, but also cardiovascular system leading to different COVID-19-associated vasculopathies. Venous and arterial thromboembolic events have been frequently described among hospitalized patients with COVID-19 and inflammatory vasculopathic changes have also been observed. Several of the reported COVID-19 associated vasculopathies exhibit differences on epidemiology, clinical characteristics and outcome compared to non-COVID-19 types. This review focuses on the epidemiology, clinical, diagnostic and therapeutic characteristics as well as outcome data of COVID-19 associated thromboembolic events and inflammatory vasculopathies, elaborating similarities and differences with non-COVID-19 cohorts.

## Introduction

**Fig. 1:**
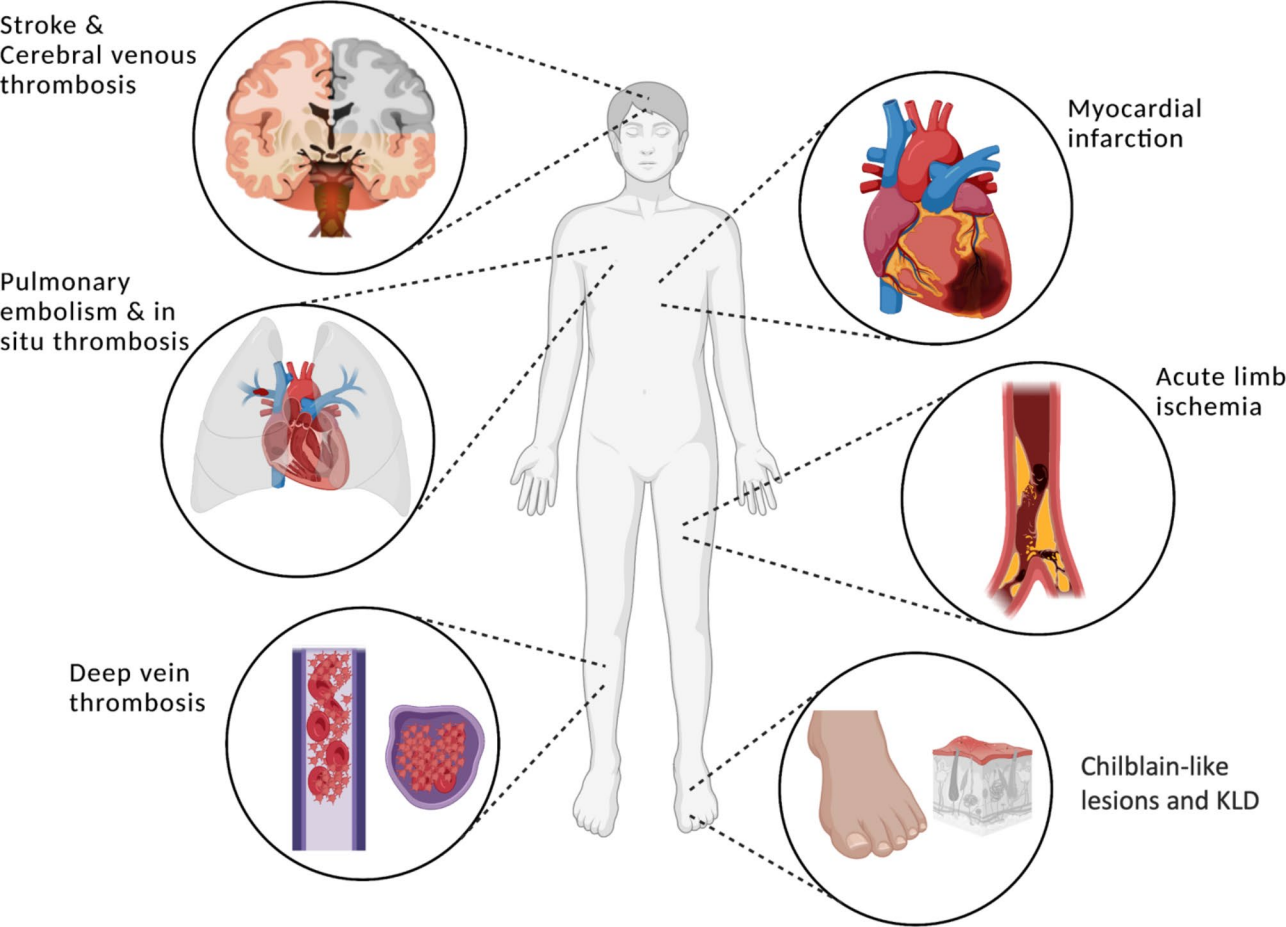
Common clinical manifestations of COVID-19 associated vasculopathies. The widespread procoagulant state and proinflammatory milieu due to SARS-CoV-2 involve the entire vasculature with thromboembolic and vasculitic complications. Figure created with BioRender.com Abbreviations: KLD: Kawasaki-like disease

In the end of 2019, severe acute respiratory syndrome-coronavirus 2 (SARS-CoV-2) spreads rapidly worldwide leading to the coronavirus disease 2019 (COVID-19) with various clinical manifestations ranging from asymptomatic cases to fatal acute respiratory distress syndrome. COVID-19 has been initially defined as a contagious disease affecting primarily the upper and lower respiratory tract. However, at a later stage in the pandemic, non-respiratory symptoms and the involvement of other organ systems than respiratory system were also observed in COVID-19. COVID-19-associated vasculopathies, including venous (VTE) and arterial thromboembolism (ATE) and to a minor extent also vasculitic changes, have been frequently described among patients with COVID-19, which potentially complicate the clinical course and increase mortality (Fig. 1) [1–3]. COVID-19 associated vasculopathies seem to arise by complex interactions between endothelial cells, coagulation and immune system after SARS-CoV-2 infection with subsequent endothelial dysfunction, immunothrombosis and formation of neutrophil extracellular traps (NETs). Thus, a hypercoagulable and pro-inflammatory state is promoted although the exact pathophysiological mechanisms need to be elucidated [4–7]. Since COVID-19 associated vasculopathies may complicate the clinical course, significant efforts were made to investigate clinical characteristics of these complications. Additionally, different anticoagulation approaches have been evaluated in various trials. This review gives an overview of epidemiological, diagnostic, therapeutic and prognostic aspects of COVID-19 associated vasculopathies.

### COVID-19 associated VTE

The frequency of COVID-19 associated VTE ranged from 13 to 14.7% according to two meta-analyses of observational studies with a reported pooled prevalence of pulmonary embolism (PE) of approximately 8% and of deep vein thrombosis (DVT) of approximately 11%, respectively. In addition, both studies revealed significantly higher VTE rates in patients admitted to intensive care unit (ICU) compared to a mixed cohort or to non-ICU patients (23.2% vs. 9% and 31% vs. 7%, respectively) [8, 9]. Contradicting the observation of higher DVT than PE rates, the meta-analysis by Kollias et al. [10] demonstrated a higher prevalence for PE in COVID-19. Screening approaches led to a significant increase of VTE detection rates with reported prevalence rates of 37% for PE and of 40% for DVT in COVID-19 patients admitted to ICU [9]. These data indicate that ICU patients with COVID-19 may benefit from screening approaches for VTE. While pre-COVID-19 data reported a DVT prevalence rate of 60.1% in patients with acute PE, this prevalence was with 11% lower in COVID-19 patients with PE [11, 12]. The results support the assumption of additional in-situ thrombus formation within pulmonary vessels, potentially triggered by immunothrombosis [6, 13]. Despite deviating results regarding the elevated risk of VTE in mixed COVID-19 cohorts vs. non-COVID-19 patients, ICU data revealed an increased risk for COVID-19 patients throughout several studies with three to six times higher VTE rates compared to a non-COVID-19 ICU cohort [1, 14–18]. Moreover, PE rates were higher in COVID-19 compared to H1N1 influenza, SARS or middle east respiratory syndrome infections [19]. Another venous thrombotic disease with increased prevalence rates in hospitalized COVID-19 patients compared to conventional cases represents cerebral venous thrombosis (CVT). According to a meta-analysis, pooled data estimated a frequency of eight cases per 10,000 in hospitalized COVID-19 patients, thus exceeding the anticipated rate of five cases per million of conventional cases from a pre-Covid era. Notably, the percentage of CVT among all cardiovascular events associated with COVID-19 was approximately 4% [20, 21]. Main risk factors for COVID-19 associated VTE include elevated D-dimer levels, male gender and concomitant cancer, while associations with age and obesity revealed divergent results. Additionally, coronary artery disease, Hispanic or Black ethnicity and HIV infection also increased the risk for VTE [12, 22–24].

#### COVID-19 associated PE and DVT

Despite a common occurrence in COVID-19, only a few data are available that investigate signs and symptoms of COVID-19 associated PE and DVT. Data of a prospective registry including patients with symptomatic and confirmed acute VTE in COVID-19 reported similar symptoms of PE as in conventional PE. 20% of patients with COVID-19 associated PE suffered from hypotension with systolic blood pressure < 100 mmHg or requirement of vasopressors and 18% had a tachycardia (heart rate > 110/min) [25]. However, symptoms of COVID-19 associated pneumonia are often similar to those of PE, including dyspnea, tachypnea, tachycardia, cough or chest pain. Thus, the clinical diagnosis of PE is potentially obscured and complicated. Of note, 88% of reported patients received thromboprophylaxis at the time of VTE diagnosis, which highlights the excessive thrombogenicity in moderate and severe COVID-19 cases [25]. Symptoms of DVT in COVID-19 were also similar to those of conventional DVT, including lower limb pain or edema as the most frequently described symptoms [26, 27]. In contrast, typical locations of COVID-19 associated PE and DVT differed from conventional PE and DVT. Most patients in a non-COVID-19 cohort presented with central PE, while segmental and subsegmental PE were predominant in COVID-19 patients [17, 28–30]. While proximal lower limb DVT occurs predominantly in non-COVID-19 cases, data of COVID-19 patients varied with similar rates of proximal and distal DVT, although a predominance of distal DVT was observed [26, 31]. An elevation of D-dimer level is a common finding in COVID-19, but does not necessarily indicate concomitant VTE. However, concomitant VTE in COVID-19 was often accompanied by markedly increased D-dimer levels. According to one study, 72.3% of DVT cases presented with D-dimer levels > 3000 ng/mL, and levels > 5000 ng/mL were robustly related to DVT (OR 19.44) [26]. Whyte et al. [32] detected even higher D-dimer levels in PE patients with a median value of 8000 ng/mL. Furthermore, C-reactive protein (CRP) levels were also significantly elevated in both PE and DVT while prothrombin time was comparable to patients without VTE [26, 32]. However, guidelines on the diagnostic approach for VTE in COVID-19 recommend against D-dimer and biomarker-based management in general [33, 34]. Instead, the diagnostic approach for VTE in COVID-19 patients should be based on clinical features which may indicate PE or DVT, such as an acute lower limb edema, erythema or pain, abrupt and unexplained clinical deterioration, hypoxemia, or right ventricular dysfunction. If any of these features is observed, computed tomography (CT) of the pulmonary arteries or compression ultrasonography of the veins should be performed for the detection of VTE [33, 34]. Echocardiography in COVID-19 patients with PE revealed pulmonary artery pressure > 40 mmHg in 36% and right ventricular dysfunction was observed in 33% [25]. Implementation of established therapeutic guidelines for anticoagulation in PE and DVT is recommended in confirmed cases with initial administration of therapeutic low-molecular-weight heparin (LMWH) and continuation of anticoagulative therapy by switching to a direct oral anticoagulant for at least three months [34]. Outcome studies revealed that COVID-19 cohorts with DVT seem to have an increased mortality risk compared to COVID-19 cases without DVT (40.3% vs. 14.9%) [35]. However, mortality rates between patients with COVID-19 associated DVT and non-COVID-19 patients with DVT were similar (17% vs. 19%, respectively) [36]. The risk of in-hospital death in patients with PE was significantly higher in a COVID-19 cohort compared to a non-COVID-19 group (12.8% vs. 5.3%) [37].

#### COVID-19 associated CVT

COVID-19 symptoms typically preceded CVT-related signs in 90% of cases, and neurological signs and symptoms seemed to be analogous to conventional CVT. Headache was the most commonly described symptom (85%), followed by epileptic seizures (42%), and altered mental status (28%), while encephalopathy, focal signs, and hemiparesis also occurred as main presenting complaints [20, 38]. COVID-19 associated CVT occurred preferably in the transverse sinus (65%), while the sigmoid sinus and superior sagittal sinus were affected in approximately 45%. Moreover, COVID-19 associated CVT occurs frequently within the deep cerebral venous system and multiple cerebral venous vessel involvement is predominately observed. Additionally, hemorrhagic lesions were detected in 42% [20, 38]. Similar to COVID-19 associated PE and DVT, data of laboratory parameters revealed elevated D-dimer and CRP levels in most COVID-19 associated CVT cases. Lymphopenia was another commonly reported laboratory finding [20, 39]. Neither diagnosis nor therapy of COVID-19 associated CVT differ from conventional CVT cases, as no specific guidelines have been established so far. Therefore, CT or magnetic resonance imaging of cerebral sinuses represent the first-line imaging techniques, and administration of anticoagulative drugs represents the first-line therapy [20, 39, 40]. Outcome data of COVID-19 associated CVT revealed higher rates of in-hospital mortality (25–40%) compared to non-COVID-19 patients with CVT (6%). Especially, parenchymal hemorrhage was associated with adverse outcome [20, 38, 41].

### COVID-19 associated ATE

Compared to VTE, ATE seems to occur less frequently in COVID-19 with a reported pooled frequency of 2.0% (95% prediction interval [PI], 0.4–9.6%) in hospitalized COVID-19 patients [2]. In this meta-analysis including a pooled cohort of more than 100,000 patients, myocardial infarction occurred in 0.8% (95% PI 0.1–8.1%), ischemic stroke in 0.9% (95% PI, 0.3–2.9%) and acute limb ischemia (ALI) in 0.2% (95% PI, 0.0-4.2%). Other ATE occurred in 0.5% (95% PI, 0.1-3.0%). Interestingly, the PIs for overall ATE frequency and myocardial infarction are quite large, which may be due to heterogeneity of the included studies and the different definitions for myocardial infarction. Another explanation for this finding may be the lower virulence and pathogenicity of new viral lineages, which was also reported by Candeloro et al. [2], but also the use of antithrombotic prophylaxis during hospitalization, as the rate of ATE decreased from 2020 to 2022 [42, 43]. Rates of hemorrhagic stroke seem to be less common than of ischemic stroke in COVID-19, but the percentage of hemorrhagic stroke is eventually higher than in non-COVID-19 cases (28:72% vs. 13:87%) [44, 45]. COVID-19 associated lower limb ischemia seems to be more frequent than upper limb ischemia, as only case reports of COVID-19 associated upper limb ischemia are available to the best of our knowledge [2, 46]. However, incidence rates of ATE in COVID-19 seem to be comparable to those in non-COVID-19 viral pneumonia, assuming that COVID-19-specific immunothrombotic mechanisms and endothelial dysfunction may have less impact on the arterial system than on the venous system [2]. Reported risk factors for the development of COVID-19 associated ATE are age, male gender, Hispanic ethnicity, preexisting arterial hypertension, diabetes, metabolic syndrome, and coronary artery disease as well as D-dimer levels > 500 ng/mL. Pre-treatment of statins may be a protective factor against ATE [22, 47]. Additionally, COVID-19 severity might also serve as a risk factor for the occurrence of ATE, as higher incidence rates of overall ATE (12%), myocardial infarction (8%), ischemic stroke (3%), and limb or mesenteric ischemia (2.5%) were reported in a systematic review by Jenner et al. [48] including more than 2900 patients treated on ICU.

#### COVID-19 associated myocardial infarction

Clinical features of COVID-19 associated acute coronary syndrome (ACS) seem to differ partially compared to non-COVID-19 ACS. Higher prevalence of atypical symptoms, such as dyspnea and syncope, than typical chest pain was observed in COVID-19 in comparison to a pre-COVID-19 cohort with ST-elevation myocardial infarction (STEMI), although chest pain was still a common symptom with up to 60% [49–51]. Additional data revealed that COVID-19 patients with STEMI and non-STEMI (NSTEMI) had higher rates of cardiogenic shock, heart failure and malignant arrhythmia compared to non-COVID-19 patients with ACS, while cardiac arrest was comparable between both cohorts [49, 52, 53]. Of note, one study revealed significantly higher rates of very late STEMI presentation (> 12 h from symptom onset) in the COVID-19 cohort compared to a control group [54]. Considerable are the findings of different studies postulating that the fear of COVID-19 exposure on the one hand and inadequate avoidance of medical institutions to prevent overburdening the system on the other hand led to delayed consultations [55–58]. Hence, this delay from symptom onset to seeking help likely contributed to higher rates of cardiogenic shock and heart failure. These reasons, however, also apply to ACS patients without COVID-19. In COVID-19, the additional prothrombotic and proinflammatory state might aggravate myocardial injury and oxygen mismatch and may explain the subsequent more pronounced ischemia and worse outcome [54, 59–61]. Interestingly, the clinical characteristics of COVID-19 associated STEMI seemed to change during the pandemic, as evaluated by Garcia et al. [62]. This study revealed that patients presented significantly more often with typical ischemic symptoms and with a trend to lower cardiogenic shock rates in 2021 compared to 2020. Changes of the electrocardiogram (ECG) were comparable among ACS patients with and without COVID-19 [51, 54]. Levels of high-sensitivity troponin, creatine kinase, and N-terminal pro-B-type natriuretic peptide were significantly elevated in STEMI patients with COVID-19 compared to STEMI patients without COVID-19, while there were no significant differences for those biomarkers between NSTEMI patients with and without COVID-19 [54, 63]. Additionally, STEMI and NSTEMI patients with COVID-19 revealed higher levels of lactate dehydrogenase (LDH) and CRP compared to non-COVID-19 cases [54]. Analyses of coronary angiographies revealed a higher thrombus burden, elevated stent thrombosis rates, and greater incidence of multiple thrombotic culprit lesions in COVID-19 patients [52, 63]. Due to diagnostic similarities of COVID-19 associated ACS with only some specific differences to non-COVID-19 ACS, the European Society of Cardiology has published a guidance paper for diagnosis and management of cardiovascular disease during COVID-19 pandemic in 2022 [64, 65]. Regarding these recommendations, the ECG diagnostic criteria for all cardiac conditions also relate to COVID-19 cases and the measurement of cardiac troponin is suggested in all cases with suspected ACS, even if troponin levels are notably elevated in severe COVID-19. Additionally, according to another joint consensus statement, COVID-19 patients with signs of concomitant acute myocardial infarction, including classic symptoms and ECG findings, might benefit from additional noninvasive imaging [66]. Therefore, the use of cardiac ultrasound to assess the cardiac function and to detect wall motion abnormalities is endorsed. These results can further support a STEMI diagnosis and evaluate the need for fibrinolysis reperfusion. Both guidance papers stated that the same treatment approach is indicated in COVID-19 patients with STEMI as for non-COVID-19 patients regarding primary percutaneous coronary intervention (PCI). Urgent fibrinolysis in COVID-19 patients with STEMI at a referral hospital is recommended, as well as a subsequent transfer for rescue PCI if indicated. COVID-19 patients with NSTEMI are also managed accordingly to non-COVID-19 cases [65, 66]. Comparing STEMI treatments in cases with and without concomitant COVID-19, door-to-balloon times were markedly increased in infected subjects. The mean door-to-balloon time was more than eight minutes longer and COVID-19 patients received primary PCI less often compared to controls (71% vs. 93%) [49, 55, 67]. These aspects may also be a reason for the significantly higher mortality in patients with COVID-19 associated STEMI compared to subjects without COVID-19 [49, 54, 67]. In the study by Garcia et al. [62], a reduction of door-to-balloon times was observed together with a decline in mortality in COVID-19 patients with STEMI when comparing the year 2020 with 2021, whereas no significant differences were detected regarding PCI rates between SARS-CoV-2 positive STEMI patients in 2020 and 2021. In contrast, there were no significant differences in mortality if COVID-19 patients with NSTEMI were compared to non-COVID-19 NSTEMI patients [54]. However, it should be mentioned that the pandemic had adverse effects on all patients with ACS, regardless of their COVID-19 status, resulting in a marked reduction in PCI and longer door-to-balloon times compared to a pre-COVID-19 era [68]. Moreover, screening for SARS-CoV-2 was always indicated where available due to infection control measures before primary PCI could proceed. Therefore, dedicated Covid-catheter labs and ICU isolation beds had to be available [69]. These logistical aspects likely have contributed to longer door-to-balloon times and increased mortality in patients with COVID-19 presenting with STEMI.

#### COVID-19 associated stroke

COVID-19 associated ischemic stroke seems to have a predominance of large vessel involvement compared to small vessel disease, which was present in only 2-4.4% of COVID-19 cases while pre-COVID-19 stroke data attribute 20% of stroke cases to small vessel disease [70–73]. In contrast, the rate of hemorrhagic strokes did not differ between COVID-19 and non-COVID-19 patients during the pandemic [74]. Vogrig et al. [73] detected multi-territory involvement and affection of otherwise uncommonly involved vessels as potential typical features of COVID-19 associated stroke. According to some studies, which compared COVID-19 patients with stroke patients before the pandemic, cryptogenic stroke rates were slightly increased in COVID-19 with reported rates of 35–40% [71, 73, 75]. In contrast, data from the “Global COVID-19 Stroke Registry” revealed a non-significant decrease of cryptogenic strokes in COVID-19 when compared to contemporaneous non-COVID-19 patients. Moreover, multi-territory involvement was comparable between COVID-19 and controls [72]. Studies demonstrated severe neurological deficits in patients with COVID-19 associated ischemic stroke and presenting with significantly elevated NIH stroke scale (NIHSS) scores in relation to non-COVID-19 associated stroke patients (median NIHSS 13 vs. 6 according to one study [72, 74]. Of note, ischemic stroke was associated with additional thrombotic events in about 25% of COVID-19 patients [73, 76]. The most common neurological findings in acute COVID-19 associated stroke were motor deficits, dysarthria, and sensory deficits [77, 78]. Altered level of consciousness, aphasia and facial paresis were additional frequently observed symptoms, and COVID-19 patients had more often seizures [74, 78]. Alterations of biomarkers in COVID-19 associated ischemic stroke included prolonged activated partial thromboplastin time and elevated CRP, fibrinogen and D-dimer levels, while data on LDH levels are divergent [73, 79]. Due to limited existing data on the specific diagnostic and therapeutic management in COVID-19 associated stroke, established stroke guidelines were also used in COVID-19 cases [80, 81]. CT imaging is used in suspected stroke, and systemic fibrinolysis or mechanical thrombectomy are performed in confirmed stroke cases if indicated. Studies comparing treatment strategies between stroke patients with and without COVID-19 revealed no differences in the frequency of performed systemic fibrinolysis or mechanical thrombectomy [82]. However, some data indicated a greater risk of intracranial hemorrhage after administration of recombinant tissue plasminogen activator (rtPA) in COVID-19 patients with elevated CRP and D-dimer levels, which are commonly increased in COVID-19 [83, 84]. One explanation for the increased risk of intracranial hemorrhage may be the fact that rtPA is hepatically metabolized and hepatic parameters are also commonly elevated in COVID-19 [73]. Although hemostasis and properties of clot formation can be safely measured by thromboelastography, also in acute liver injury, no decision-making is recommended for or against systemic fibrinolysis depending on laboratory markers in COVID-19 so far [84, 85]. In contrast, the performance of mechanical thrombectomy in COVID-19 associated stroke revealed lower rates of successful recanalization, while no difference was shown in treatment duration and symptom-to-treatment times [72]. In general, COVID-19 patients with stroke exhibited higher rates of symptomatic intracerebral hemorrhage, symptomatic subarachnoid hemorrhage, and 24-hour mortality following intravenous thrombolysis as well as endovascular treatment in relation to a contemporaneous non-COVID-19 cohort [72]. Another study evaluating outcome data of patients with COVID-19 associated stroke revealed a significantly elevated in-hospital mortality compared to COVID-19 patients without stroke (19.4% vs. 6.2%) with an increased risk for the development of cerebral edema, intracerebral hemorrhage, and myocardial infarction [82, 86, 87]. Additionally, COVID-19 associated stroke was associated with higher rates of ICU admission and intubation compared to stroke patients without COVID-19 [88]. Moreover, Hernández-Fernández et al. [89] revealed that COVID-19 associated stroke patients may exhibit longer hospitalization rates, slower recovery times and unfavorable functional prognosis evaluated by the modified Rankin Scale in over 64% of cases. These outcomes are supported by the “Global COVID-19 Stroke Registry” which revealed higher 3-month mortality rates, worse 3-month mortality modified Rankin scale shift as well as 3-month favorable outcomes in their COVID-19 cohort [72].

#### COVID-19 associated limb ischemia

As mentioned above, COVID-19 associated ALI is a rare finding among patients with COVID-19 [2]. In the majority, ALI was diagnosed subsequently to SARS-CoV-2 infection while 27% presented to hospital with ALI symptoms alone, assuming a quite high rate of asymptomatic SARS-CoV-2 infection in ALI [90]. COVID-19 associated ALI most commonly affects femoro-popliteal arteries, followed by tibial arteries [91, 92]. Additionally, Goldman et al. [93] found higher rates of COVID-19 associated ALI in more proximal regions as well as a greater clot burden. Of the reported studies, leg pain and leg discoloration were the most frequent symptoms of COVID-19 associated ALI, which is comparable to non-COVID-19 associated ALI. Also the frequency of the Rutherford classification grading used in ALI is comparable between COVID-19 and non-COVID-19 revealing that Rutherford IIa and IIb are most commonly observed grades [91–94]. Except of an increase of D-dimer levels during hospitalization in COVID-19, which may be an indicator of ALI, there is limited significance of other laboratory markers for the diagnosis of ALI [95, 96]. Recommendations of the European Society for Vascular Surgery on an adjusted management approach in COVID-19 associated ALI suggest a preferential use of CT angiography for the detection of ALI, but with the advice to extend the imaging from the aortic arch to the feet or hands due to the anatomically more extensive disease. Following verified ALI diagnosis, the standard management approach also applies to COVID-19 patients including adequate analgesia, rehydration and administration of heparin. Open surgery and endovascular treatment are the definite therapeutic options and thromboembolectomy is the most used technique, while hospitalized COVID-19 patients, especially those with severe disease, might benefit from endovascular techniques as a less invasive procedure [90, 96, 97]. In contrast, a systematic review by Galyfos et al. [98] revealed that medical treatment was selected as first-line therapy in more than 40% of included COVID-19 associated ALI patients. This treatment option led on the one hand to a non-significantly elevated amputation risk, but on the other hand to an increased mortality compared to COVID-19 associated ALI patients receiving any intervention. Moreover, studies reported that 14–23% of COVID-19 patients already receiving anticoagulation at the time of ALI diagnosis developed rethrombosis after revascularization despite full heparin-based anticoagulation [92, 99–101]. Similar results were also reported by another systematic review, in which 13% of intervened COVID-19 associated ALI patients required a re-intervention due to persistent or recurring limb ischemia. Additionally, the technical success rate in these patients was low with 68%. Of note, 19% of COVID-19 associated ALI patients were ineligible for surgical or endovascular procedures due to critical status [90]. Amputation and mortality rates of COVID-19 associated ALI cohorts ranged between 8 and 25% and between 29 and 38%, respectively, which are higher compared to non-COVID-19 patients with ALI [90, 93, 96, 98, 102, 103]. Additionally, mortality and amputation rates were lower in COVID-19 associated ALI patients who had symptoms limited to the leg without other systemic or respiratory COVID-19 manifestations [93].

#### COVID-19 associated other ATE

Other COVID-19 associated ATE include mesenteric ischemia, splenic, and renal arterial infarctions. All three conditions are, however, rarely reported in the literature and mostly only in case reports [104–107]. While conclusive data on mesenteric ischemia are yet limited and derived at least from comprehensive or systematic reviews including case reports and small case series, conclusive data on splenic or renal arterial infarctions are to the best of our knowledge missing and cannot be reflected in this review. Another limitation of COVID-19 associated mesenteric ischemia is the fact that the derived studies differentiated only partially or not between ATE or VTE as the origin of mesenteric ischemia [108–111]. Reported rates of detected thrombi in visceral arteries in COVID-19 associated mesenteric ischemia range from 15 to 38%, suggesting a predominant small vessel affection [109, 111]. Of those reported COVID-19 patients with arterial mesenteric ischemia, abdominal pain, vomiting and nausea were frequently observed symptoms, and abdominal distension was a common finding in physical examination. CT was the imaging method of choice for its detection [108–111]. No differentiation was made between arterial and venous mesenteric ischemia in the reported studies regarding biomarkers, therapy or outcome. Therefore, only pooled results for arterial and venous mesenteric ischemia can be reflected in this review. D-dimer, CRP and leukocytes were commonly elevated but did not correlate with outcome [109, 111]. COVID-19 associated mesenteric ischemia was typically treated by initial administration of heparin followed mostly by laparotomy with ischemic bowel resection, while endovascular interventions were only rarely performed (< 5%) [109, 111]. Reported mortality rates of COVID-19 associated mesenteric ischemia ranged from 34 to 54% [108–111].

## COVID-19 associated vasculitis

Epidemiological data of COVID-19 associated vasculitides are scarce as specific vasculitides other than COVID-19 associated endotheliitis are a rare manifestation in COVID-19 [112]. The most common forms include COVID-19 associated Chilbalin-like lesions (CLL), commonly called colloquially as ‘Covid toes’, and COVID-19 associated Kawasaki-like disease (KLD) [113]. COVID-19 associated CLL seem to occur more frequently in Europe and North America, in adolescent patients between 15 and 19 years without predominant sex, and in patients with a low body-mass index [114–116]. However, definite prevalence or incidence rates are still missing with only proposed incidence rates ranging widely from 0.2 to 20% of reported cases [115]. Parts of the foot are more commonly affected by COVID-19 associated CLL than parts of the hand [116]. COVID-19 associated KLD is commonly associated with multisystem inflammatory syndrome in children (MIS-C) or pediatric inflammatory multisystem syndrome (PIMS), which represent a systemic disorder featuring fever, hyperinflammation and organ dysfunction associated with COVID-19. Specific epidemiological data for COVID-19 associated KLD are not available so far, while incidence rates for MIS-C or PIMS ranging from 0.03 to 0.07% with a reported median age of eight years [117–119]. MIS-C can occur as KLD but symptoms comparable to toxic shock syndrome or bacterial sepsis may be also possible, and coronary dilatations and aneurysms may also occur in MIS-C without meeting the criteria for Kawasaki disease [119–121]. Due to this overlap, a strict differentiation between KLD and MIS-C or PIMS is hardly possible, also in literature. Clinical features of COVID-19 associated KLD compared to pre-outbreak cohorts of Kawasaki disease revealing that gastrointestinal and neurological symptoms, myocarditis, and shock syndrome occurred more frequently while coronary dilatations and aneurysms are less common in KLD [121–124]. Evidence of potential COVID-19 association on other specific vasculitides, including ANCA-vasculitis, giant-cell arteritis, immunoglobulin A vasculitis or Goodpasture syndrome, are largely missing and only some case reports and case series mentioned a potential linkage between SARS-CoV-2 infection and their occurrence [125–128]. Specific diagnostic, therapeutic and prognostic aspects of COVID-19 associated vasculitis are yet reported for CLL and KLD, while the respective aspects of other vasculitides with a reported potential COVID-19 association are comparable to non-COVID-19 patients.

In patients with COVID-19 associated CLL, abnormal D-dimer levels and antinuclear antibodies were found in 14.8% and in 11.5% while abnormalities of inflammatory parameters like ferritin, CRP or lymphocytes were found only sporadically [116]. COVID-19 associated CLL seem to be a benign and self-limited disorder as only 15.5% of patients had received any form of therapy, ranging from topical corticoids, heparin and nitroglycerin to oral corticoids, subcutaneous heparins and tocilizumab according to a meta-analysis [116]. However, digital necrosis with histopathologic features of vasculitis have also been reported by case reports while all of these reported cases occurred in severe COVID-19 cases [129–131]. Therefore, it remains elusive if vasculitic processes were the exclusive trigger of necrotic acral lesions or if digital necrosis occurred due to an interaction of immunomodulated thrombotic disturbances, which are frequently seen in severe COVID-19 [132]. COVID-19 associated KLD patients revealed higher levels of CRP, ferritin, procalcitonin, alanine aminotransferase, lipase, troponin and creatinine and lower levels of lymphocytes, hemoglobin and sodium, while fibrinogen was inconsistently elevated [121, 124]. The treatment regimen for COVID-19 associated KLD did not differ to classic Kawasaki disease and there is no consensus so far which therapeutic regimen is ideal or better suited. Thus, intravenous immunoglobulin with or without aspirin and additional immunosuppressive agents, including steroids, infliximab or tocilizumab, are administered depending on the clinical course and clinicians’ preference. However, patients with COVID-19 associated KLD received more often additional corticosteroids in combination with immunoglobulins [122, 124]. Short-term outcome of COVID-19 associated KLD seems to be favorable regarding cardiac function and coronary aneurysm regression, while long-term cardiac outcome data or specific mortality rates for KLD are scarce [124, 133]. In pediatric and adolescent MIS-C, a mortality rate of 1.76% have been reported [122].

## Anticoagulation in COVID-19

During the pandemic, various antithrombotic treatment and management approaches have been evaluated and discussed. As the occurrence of VTE dominated, different trials primarily investigated the outcomes of hospitalized COVID-19 patients without suspected or with confirmed VTE depending on prophylactic or therapeutic anticoagulation regimens [134, 135]. One main finding was that all hospitalized patients with COVID-19 receive VTE prophylaxis, unless contraindicated (e.g. active bleeding) [136]. Typically, heparins are administered, with LMWH being the preferred agent. In the case of a history of heparin-induced thrombocytopenia, fondaparinux might serve as an alternative. Patients already receiving anticoagulants before hospital admission should continue their medication unless contraindications arise. Though, a switch to shorter acting agents such as LMWH might be indicated [136, 137]. In non-critically ill, hospitalized patients, therapeutic heparin doses are recommended over prophylactic doses, resulting in increased organ support-free days [134, 135, 137, 138]. However, therapeutic heparin dose did not reveal significant advantages regarding in-hospital mortality or hospitalization length, but was associated with a slightly elevated risk of major bleeding [135]. In patients admitted to ICU without suspected or documented VTE, prophylactic heparin doses are preferred over therapeutic or intermediate heparin doses due to lack of significant advantages of therapeutic anticoagulation regarding all-cause mortality or organ support-free days [134, 136–139]. Thus, in the case of deteriorating patients with subsequent ICU admission, initial treatment with therapeutic heparin doses should be switched to prophylactic doses unless VTE is confirmed [137].

All included panels recommend against the prescription of anticoagulants in non-hospitalized COVID-19 patients with normal risk for VTE and no indication for post-discharge prophylaxis when no VTE was detected [136–138]. However, in high-risk individuals, post-discharge thromboprophylaxis with 10 mg rivaroxaban daily significantly reduced the composite endpoint (VTE, symptomatic ATE, any fatal cardiovascular event) according to one trial [140]. Moreover, no panel recommend the administration of antiplatelet therapy, neither in hospitalized patients nor in outpatients, as different trials concluded no advantages regarding a reduction in organ support-free days, in-hospital mortality, or risk of progression to mechanical ventilation [137, 141–144]. Finally, it should be addressed that the current dominance of SARS-CoV-2 omicron variant and its subtypes could change these recommendations due to milder and potentially less thrombogenic courses [136].

## Conclusion

Epidemiological data attribute hospitalized COVID-19 patients an increased risk for COVID-19 associated VTE, also if compared to other viral infectious diseases. Studies emphasized notably higher rates of VTE in COVID-19 ICU cohorts, especially if a systematical screening approach was used. As screening approaches led to even higher detection rates of VTE, and data revealing a predominance of smaller vessel affection, the question arises whether these examinations actually caused the increased rates of otherwise occult thrombi. Consequently, screenings in other infections, such as influenza, might also detect higher rates of occult and asymptomatic thrombi. However, in the case of COVID-19 patients being transferred to ICU because of clinical deterioration, the screening approach could unveil its cause and is therefore reasonable.

COVID-19 associated ATE and vasculitic disorders seem to occur with a comparable extent like in other viral infectious diseases, although frequencies rise with severity of COVID-19. Of interest is the finding that PE was comparable or even more prevalent to DVT in some studies, suggesting a role of in-situ pulmonary thrombi formation, which may be a disease-specific feature in COVID-19. In-situ pulmonary thrombi formation may be driven by endothelial dysfunction, immunothrombotic processes and formation of NETs [4–7]. However, these pathways may affect the arterial circulation to a lesser extent as the frequency rates were comparable to non-COVID-19 viral pneumonia, except in severe COVID-19 cases [2, 48]. Furthermore, these pathophysiological pathways seem to affect, at least in the venous system but not in the arterial system, predominantly smaller-sized vessels, as incidence rates of sub-/segmental PE and distal DVT were higher [28–31, 71, 73, 91, 92]. Additionally, the microcirculation may be also affected to a certain extent as COVID-19 associated CLL is not uncommon, while other forms of vasculitides are only anecdotally described, except for KLD [115]. Despite some epidemiological differences, many clinical aspects of COVID-19 associated vasculopathies revealed comparable results to non-COVID-19 patients (Table 1). Symptoms of the above-mentioned COVID-19 associated vasculopathies did not differ relevantly compared to the respective conventional disorders, as only COVID-19 associated myocardial infarction had a higher prevalence of atypical symptoms [49–51]. Similarly, determined risk factors, diagnostic approaches including biomarkers, and treatment options for the respective COVID-19 associated vasculopathies are also comparable to conventional vasculopathies. High efforts were made to establish reliable biomarkers or at least cut-off values for COVID-19 associated VTE or ATE, but without desired results. Especially, D-dimer was a frequently investigated parameter and some results were conclusive [26, 32]. However, this parameter is not a robust marker for COVID-19 associated VTE or ATE as severe COVID-19 disease without evidence of VTE or ATE also exhibits substantially increased D-dimer levels. Other conventional biomarkers, such as CRP, have limited significance and specific cut-off values are lacking so far to discriminate reliably between severe COVID-19 and actual VTE or ATE. Therefore, biomarker-based management in COVID-19 is not recommended so far. Moreover, although some international societies already published COVID-19 specific diagnostic and therapeutic recommendations, these recommendations are mostly identical to those of the respective conventional vasculopathies, with only slight modifications [33, 34, 64, 65, 96]. Prophylactic anticoagulation in COVID-19 depends on the severity of disease, without recommendation for non-hospitalized patients at normal risk. In hospitalized non-critically ill individuals, therapeutic doses of LMWH are preferred, while critically ill patients in ICU benefit from prophylactic doses. Regarding outcome data, many COVID-19 associated vasculopathies seem to be unfavorable and may severely deteriorate the clinical course, except for DVT, CLL and KLD, while long-term data regarding cardiac complications are still missing in the latter. Especially, acute myocardial infarction and acute stroke were markedly more severe in COVID-19 patients and associated with increased mortality and worse clinical outcome despite identical treatment approaches. However, it should be mentioned that the pandemic had adverse effects on all patients with ATE, regardless of their COVID-19 status. A marked reduction of PCI and longer door-to-balloon times in COVID-19 patients with ACS compared to a pre-COVID-19 era was reported, and these logistical aspects may also attribute to other ATE [68]. Therefore, physicians should be aware of these vascular COVID-19 associated complications, especially in hospitalized and ICU-treated COVID-19 patients.

**Table 1:** Comparison of vascular complications of hospitalized COVID-19 patients to non-COVID-19 cohorts with the main differences and similarities

	DVT	PE	CVT	MI	Stroke	ALI
**Incidence**	↑	↑	↑	=	=	=
**Severity**	=	↓	↑	↑	↑	=
**Symptoms**	=	=	=	=*	=	=
**Diagnosis**	=	=	=	=	=	=
**Therapy**	=	=	=	=	=	=
**Mortality**	=	↑	↑	↑	↑	↑

=: no significant difference

*: more atypical symptoms

Abbreviations: ALI: acute limb ischemia; CVT: cerebral venous thrombosis; DVT: deep vein thrombosis; MI: myocardial infarction; PE: pulmonary embolism

Despite tremendous knowledge growth in the field of COVID-19 associated vasculopathies, the gained knowledge is still subject to limitations. Firstly, reported prevalence and incidence varied partially widely across the studies and different screening approaches for the respective VTE or ATE were used. Additionally, the increasing administration of anticoagulation in newer studies and the fact that cohorts included patients from different time points during the pandemic may substantially influence epidemiological and outcome data. Furthermore, on the one hand, several meta-analyses included mainly retrospective studies, and on the other hand, several studies on specific clinical data, including symptomatology and subtypes of COVID-19 associated vasculopathies, included only a small sample size. All of this limits the significance. Moreover, diagnostic and treatment recommendations, which originated from the initial stage of the pandemic, might be outdated, and, as most studies solely declared all-cause mortality within their cohorts, the actual proportion of thromboembolic deaths remains still elusive.

Overall, specific vascular diseases seem to occur frequently in COVID-19 which may complicate the clinical course. Although numerous studies on clinical aspects of COVID-19 associated vasculopathies are available, the gained knowledge has still limitations. Therefore, further clinical research is required for specific diagnostic and therapeutic options and to reveal robust outcome and long-term sequelae data in COVID-19 associated vasculopathies.

## Data Availability

All data generated or analysed during this study are included in this published article.

## Abbreviations

ACS: Acute coronary syndrome
ALI: Acute limb ischemia
ATE: Arterial thromboembolism
CLL: Chilblain-like lesions
COVID-19: Coronavirus disease 2019
CRP: C-reactive protein
CVT: Cerebral venous thrombosis
DVT: Deep vein thrombosis
ECG: Electrocardiogram
ICU: Intensive care unit
KLD: Kawasaki-like disease
LDH: Lactate dehydrogenase
LMWH: Low-molecular-weight heparin
MIS-C: Multisystem inflammatory syndrome in children
NETs: Neutrophil extracellular traps
NSTEMI: Non-ST-elevation myocardial infarction
PCI: Percutaneous coronary intervention
PE: Pulmonary embolism
PI: Prediction interval
PIMS: Pediatric inflammatory multisystem syndrome
rtPA: Recombinant tissue plasminogen activator
SARS-CoV-2: Severe acute respiratory syndrome-coronavirus 2
STEMI: ST-elevation myocardial infarction
VTE: Venous thromboembolism
